# Direct Factor X sequestration by systemic amyloid light‐chain amyloidosis

**DOI:** 10.1002/ccr3.1398

**Published:** 2018-01-31

**Authors:** Haruko Tashiro, Ryosuke Shirasaki, Masato Watanabe, Kazuo Kawasugi, Yoshihisa Takahashi, Naoki Shirafuji

**Affiliations:** ^1^ Department of Hematology/Oncology Teikyo University School of Medicine Itabashi‐ku Tokyo Japan; ^2^ Department of Pathology Teikyo University School of Medicine Itabashi‐ku Tokyo Japan; ^3^ Department of Pathology Graduate School of Medical Sciences International University of Health and Welfare Narita Chiba Japan

**Keywords:** Amyloid Light‐chain amyloidosis, Factor X antibody, Factor X deficiency, lymphoplasmacytic lymphoma

## Abstract

We present a lymphoplasmacytic lymphoma patient with Factor X (FX) deficiency. Despite the absence of FX inhibitor, the administration of fresh frozen plasma and anti‐inhibitor coagulant complex did not increase the FX level. The autopsy showed that massive amyloid depositions to multiple organs and FX existed in union with amyloidosis.

Amyloid light‐chain (AL) amyloidosis is a well‐recognized complication of plasma cell dyscrasias such as multiple myeloma and Waldenström macroglobulinemia, as well as B‐cell lymphomas 1, 2. Six to fourteen percent of AL amyloidosis patients develop an acquired Factor X (FX) deficiency 3, 4, 5. The direct binding/adsorption of FX onto amyloid fibrils has been thought to be a major mechanism for FX deficiency in amyloidosis. Furie et al. 6 first showed that infused FX rapidly disappeared from the circulation and distributed over the body surface in an amyloidosis patient with FX deficiency. A mechanism to account for this phenomenon was provided in a subsequent work, where the group used in vitro experiments to demonstrate that FX binds to amyloid fibrils isolated from patients with primary amyloidosis with FX deficiency 7. However, direct pathological evidence of FX binding to amyloid in patient tissues has not yet been reported.

## Case Report

**Figure 1:**
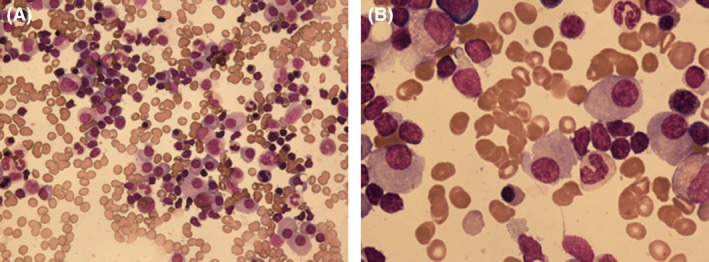
Bone marrow aspirate showing increased number of lymphocytes, plasmacytoid lymphocytes, and plasma cells. (A) May‐Giemsa staining 400X, (B) 1000X.

**Figure 2:**
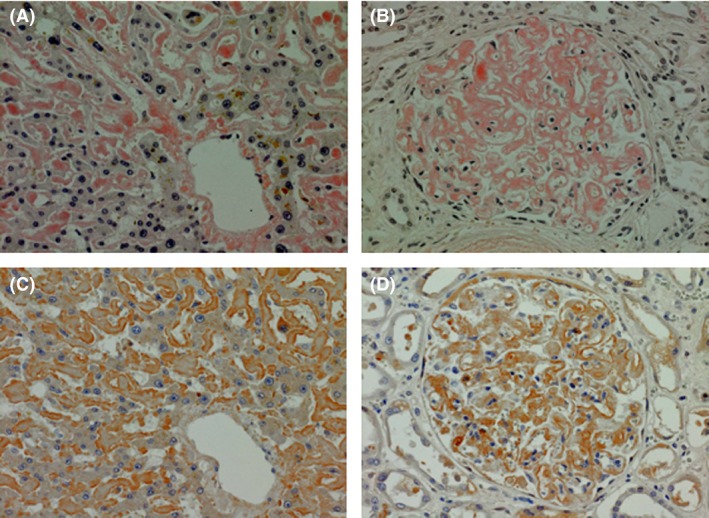
Autopsy specimens stained with Congo red (A and B) and anti‐Factor X antibody (C and D). (A) Liver, Congo red, amyloid deposition to sinusoid. (B) Kidney, Congo red, amyloid deposition mainly to mesangial matrix. (C and D) Liver and kidney stained with anti‐Factor X, showing that Factor X exists in union with amyloid.

**Figure 3:**
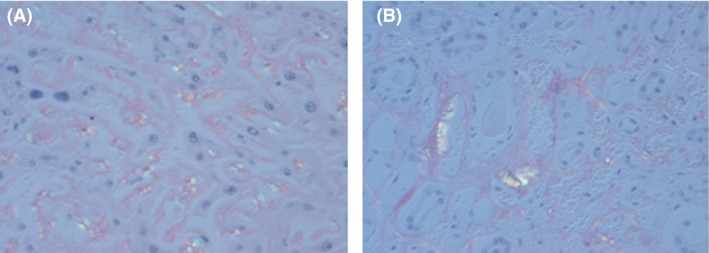
Autopsy specimens stained with Congo red under polarized light revealed the apple‐green birefringence appearance. (A) Liver and (B) Kidney.

A previously healthy 37‐year‐old woman was presented with spontaneous bleeding, para‐aortic lymphadenopathy, and splenomegaly. Her initial laboratory tests demonstrated the following: WBC 10.1 × 10^9^/L, hemoglobin 6.5 g/dL, and platelets 137 × 10^9^/L with normal LDH and normal liver function tests. While her creatinine level was within normal limits, a urine test revealed proteinuria with Bence–Jones *κ*‐type M protein. Serum immunoglobulin levels of IgG, IgA, IgM, and IgD were 887, 102, 171 mg/dL, and under detectable levels, respectively. Coagulation study revealed a normal fibrin degradation products level with prolongation of prothrombin time (PT) (34.1 sec) and activated partial thromboplastin time (APTT) (54.4 sec). The coagulation factor levels were within normal ranges except Factor X (FX) which was severely decreased (2%, normal >70%) and slight decrease in Factor IX (42%). No FX inhibitor was detected, and a cross‐mixing test revealed a deficiency pattern. A bone marrow smear showed 40% of plasmacytoid lymphocytes that were positive for CD19, CD20, and CD38 and negative for CD5, CD10, CD23, and CD56 (Fig. 1). A lymph node biopsy was not performed because of severe bleeding risk. The patient was tentatively diagnosed with lymphoplasmacytic lymphoma with acquired FX deficiency. Although we suspected that the patient had developed systemic amyloidosis, we were unable to confirm the diagnosis because her severe bleeding risk restricted us from obtaining tissues samples, with the exception of bone marrow and skin (which did not show amyloid deposition). Although she received several chemotherapeutic reagents, including steroids, cyclophosphamide, rituximab, fludarabine, cladribine, bortezomib, and thalidomide, her hepatosplenomegaly and lymphadenopathy progressed. Despite the massive administration of fresh frozen plasma and anti‐inhibitor coagulant complex, the FX level was not increased and her bleeding tendency was not recovered. Additional plasma exchanges were unable to increase her FX levels. She received allogeneic bone marrow transplantation (BMT) from her human leukocyte antigen‐matched sibling with reduced intensity conditioning. However, eventually the patient died from multi‐organ failure after BMT. An autopsy was performed, and amyloid depositions were shown in multiple organs, including liver, spleen, kidney, bone marrow, lymph nodes, adrenal glands, lungs, and heart by Congo red staining (Fig. 2A and B). The Congo red staining specimens revealed apple‐green birefringence appearance under polarized light (Fig. 3A and B). Lymphocytes in the lymph nodes were severely decreased, and no tumor cells were detected. To directly show that the intractable FX deficiency was due to the sequestration of FX by amyloid in vivo, we stained these tissues with anti‐Factor X antibody (Abcam, Cambridge, UK) as well. Indeed, FX existed in union with amyloid (Fig. 2C and D). To our knowledge, this is the first histological demonstration of direct deposition of FX onto amyloid. Our work supports the established mechanism of amyloid‐induced disappearance of FX from circulation 6, 7.

## Consent

Written informed consent was obtained from the patient' kin for publishing the case report.

## Authorship

HT, RS, KK, and NS: involved in patient management. MW and YT: provided pathological findings including Factor X staining. HT, YT, and NS: provided editing and review of the manuscript. HT: wrote the manuscript.

## Conflict of Interest

None declared.
